# IL-6 Is Not Necessary for the Regulation of Adipose Tissue Mitochondrial Content

**DOI:** 10.1371/journal.pone.0051233

**Published:** 2012-12-11

**Authors:** Zhongxiao Wan, Christopher G. R. Perry, Tara Macdonald, Catherine B. Chan, Graham P. Holloway, David C. Wright

**Affiliations:** 1 Department of Agriculture, Food and Nutritional Science, University of Alberta, Edmonton, Alberta, Canada; 2 Department of Human Health and Nutritional Sciences, University of Guelph, Guelph, Ontario, Canada; Tohoku University, Japan

## Abstract

**Background:**

Adipose tissue mitochondria have been implicated as key mediators of systemic metabolism. We have shown that IL-6 activates AMPK, a mediator of mitochondrial biogenesis, in adipose tissue; however, IL-6^−/−^ mice fed a high fat diet have been reported to develop insulin resistance. These findings suggest that IL-6 may control adipose tissue mitochondrial content in vivo, and that reductions in adipose tissue mitochondria may be causally linked to the development of insulin resistance in IL-6^−/−^ mice fed a high fat diet. On the other hand, IL-6 has been implicated as a negative regulator of insulin action. Given these discrepancies the purpose of the present investigation was to further evaluate the relationship between IL-6, adipose tissue mitochondrial content and whole body insulin action.

**Methodology and Principal Findings:**

In cultured epididymal mouse adipose tissue IL-6 (75 ng/ml) induced the expression of the transcriptional co-activators PGC-1α and PRC, reputed mediators of mitochondrial biogenesis. Similarly, IL-6 increased the expression of COXIV and CPT-1. These effects were absent in cultured subcutaneous adipose tissue and were associated with lower levels of GP130 and IL-6 receptor alpha protein content. Markers of mitochondrial content were intact in adipose tissue from chow fed IL-6^−/−^ mice. When fed a high fat diet IL-6^−/−^ mice were more glucose and insulin intolerant than controls fed the same diet; however this was not explained by decreases in adipose tissue mitochondrial content or respiration.

**Conclusions and Significance:**

Our findings demonstrate depot-specific differences in the ability of IL-6 to induce PGC-1α and mitochondrial enzymes and demonstrate that IL-6 is not necessary for the maintenance of adipose tissue mitochondrial content in vivo. Moreover, reductions in adipose tissue mitochondria do not explain the greater insulin resistance in IL-6^−/−^ mice fed a high fat diet. These results question the role of adipose tissue mitochondrial dysfunction in the etiology of insulin resistance.

## Introduction

PPAR gamma co-activator 1 alpha (PGC-1α) is a transcriptional co-activator that binds to, and co-activates transcription factors leading to the coordinated regulation of mitochondrial and nuclear encoded mitochondrial enzymes [1]. In addition to PGC-1α, the related co-activators, PGC-1β and PGC-1 related co-activator (PRC) appear to play at least somewhat overlapping roles in regulating mitochondrial enzyme gene expression [2], [3]. The role of PGC-1α in the regulation of mitochondrial biogenesis has traditionally been assessed in tissues with high oxidative capacities such as heart [2], [4], [5] and skeletal muscle [6], [7]. However, accumulating evidence points towards a key role for PGC-1α in regulating mitochondrial biogenesis in fat cells. For example, the over-expression of PGC-1α increases mitochondrial enzyme expression in human adipocytes [8], while recent work from Spiegelman's laboratory [9] has elegantly demonstrated that the adipose tissue specific ablation of PGC-1α leads to decreases in the expression of thermogenic and mitochondrial genes in that tissue.

Adipose tissue mitochondria have been suggested to be important regulators of systemic glucose homeostasis. For instance mitochondrial content is reduced in adipose tissue from obese, insulin resistant rodents [10], [11], [12], whereas thiazolidinediones induce adipose tissue mitochondrial biogenesis concomitant with increases in insulin sensitivity [12], [13]. Interestingly, when adipose tissue specific PGC-1α knockout mice are fed a high fat diet they are more insulin resistant than their WT littermates [9]. Collectively, these findings underscore the potential importance of adipose tissue mitochondria in the regulation of fuel homeostasis.

Over the past decade interleukin 6 (IL-6) has gained considerable attention as a muscle-derived hormone, or myokine (reviewed in [14]), with several groups providing evidence that IL-6 stimulates skeletal muscle fatty acid oxidation [15], [16] and glucose disposal [17]. In addition to skeletal muscle, IL-6 is also expressed in adipose tissue [18] with higher levels being reported in human abdominal versus subcutaneous depots [18]. The interstitial concentrations of IL-6 surrounding adipose tissue are orders of magnitude greater than levels in the circulation [19], [20] leading to the suggestion that adipose tissue-derived IL-6 regulates adipose tissue metabolism in an autocrine/paracrine manner [20]. Recent work from our laboratory has provided evidence that IL-6 could control adipose tissue mitochondrial content [21]. We found that IL-6 activates 5′AMP activated protein kinase (AMPK) in cultured adipose tissue, while AMPK phosphorylation was reduced in adipose tissue from whole body IL-6^−/−^ mice [21]. AMPK is a reputed regulator of PGC-1α and mitochondrial biogenesis [22] and thus it seems likely that IL-6 could control PGC-1α and mitochondrial enzymes in adipose tissue. In support of this view IL-6 has been shown to induce PGC-1α in cultured 3T3 adipocytes [23]. However, it is yet to be determined if IL-6 regulates PGC-1α, or the related co-activators PGC-1β and PRC (PGC-1 related co-activator), and mitochondrial enzymes in adipose tissue, or if this occurs in a depot-specific manner.

IL-6 deficient mice have been reported to be insulin resistant [24] and this appears to be exacerbated during maturation [25], [26] and when consuming a high fat diet [25]. Given the purported role of adipose tissue mitochondria in the regulation of systemic carbohydrate metabolism [9] and recent work linking IL-6 to the induction of adipose tissue mitochondrial enzyme gene expression [21], [23] it seems likely that reductions in adipose tissue mitochondrial content could be causally linked to the development of insulin resistance in IL-6 deficient mice when challenged with a high fat diet. This hypothesis, while attractive, is difficult to reconcile with the long held view that IL-6, either directly [27], [28], or working synergistically with other cytokines [29], [30] causes adipocyte and whole body insulin resistance [27], [28], [29], [30], [31]. Given these discrepancies the purpose of the present investigation was to further evaluate the relationship between IL-6, adipose tissue mitochondrial content and whole body glucose homeostasis. Using cultured mouse adipose tissue and whole body IL-6 deficient mice, we tested the hypothesis that IL-6 would regulate PGC-1α and mitochondrial enzyme gene expression in adipose tissue. As IL-6 secretion has been shown to be greater in abdominal compared to subcutaneous adipose [18], we postulated that IL-6 would regulate PGC-1α and mitochondrial enzymes in epididymal (an abdominal fat depot) but not subcutaneous mouse adipose tissue. Lastly, we hypothesized that IL-6 deficient mice when fed a high fat diet would be more insulin resistant and glucose intolerant that wild type controls fed the same diet and that this would be explained, at least in part, by reductions in adipose tissue mitochondrial content and respiration.

## Results

### IL-6 induces the expression of PGC-1α, PRC and mitochondrial enzymes in adipose tissue in a depot specific manner

**Figure 1:**
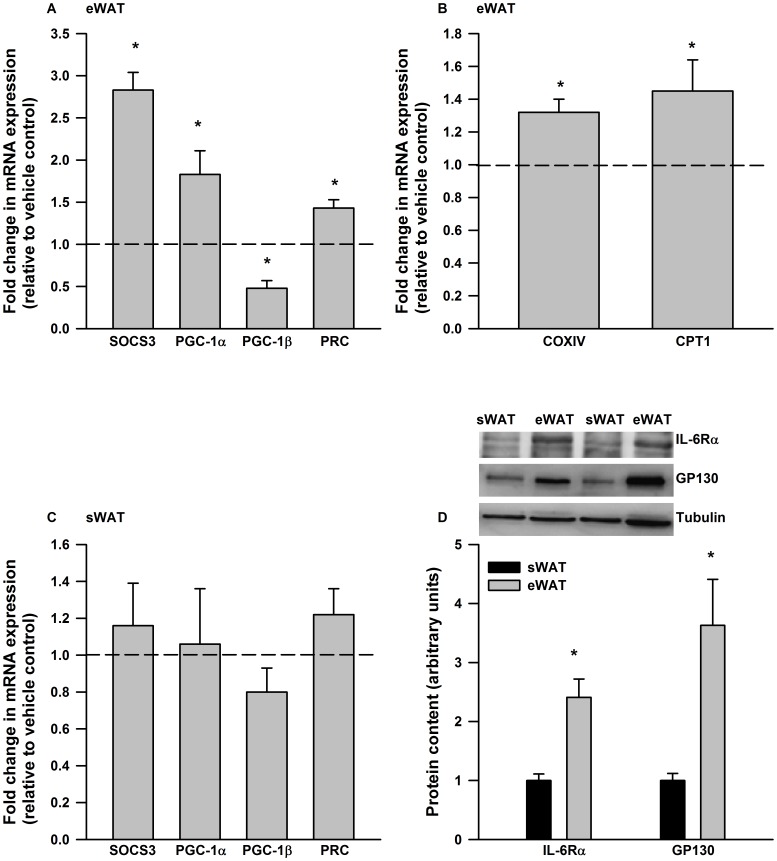
IL-6 exerts depot specific differences in gene expression in mouse adipose tissue. IL-6 treatment (6 hours, 75 ng/ml) increased the expression of A) SOCS3, PGC-1α and PRC mRNA in cultured epididymal adipose tissue (eWAT). B) A 12 hr IL-6 (75 ng/ml) treatment increased the expression of COXIV and CPT-1 in cultured eWAT. C) Cultured subcutaneous adipose tissue (sWAT) was not responsive to IL-6 treatment (75 ng/ml, 6 hours) and this was associated with D) reductions in the protein content of IL-6 receptor alpha and GP130. Data are presented as means + SE for 7 cultures, each from an individual mouse, per group and is expressed relative to the vehicle treated control culture from the same animal. For the Western blot data in D) data are presented as means + SE for 8–10 mice per group. Representative Western blots are presented above the quantified data. * P<0.05.

Previous work has shown that IL-6 induces PGC-1α mRNA expression in 3T3 adipocytes [23]. However, it is not known if IL-6 induces similar effects in adipose tissue, or if depot specific differences in the ability of IL-6 to increase PGC-1α, or the related co-activators PGC-1β and PRC, exist. To answer this question we treated cultured mouse epididymal and subcutaneous adipose tissue with IL-6 (75 ng/ml) for 6 hours. We chose this dose of IL-6 based on previous data from our laboratory [21] and others [32], which demonstrated that similar concentrations of IL-6 activated AMPK and STAT3 (Signal Transducer and Activator of Transcription 3) and induced SOCS3 (Suppressor of Cytokine Signaling 3) mRNA in cultured adipocytes and adipose tissue. Although the concentration of IL-6 that we chose to use is much higher than that typically found in the circulation (pg/ml), it is within the range of concentrations found in the interstitial space surrounding fat cells [19]. As seen in Figure 1A, IL-6 treatment resulted in significant increases in the expression of SOCS3, a transcriptional target of IL-6 signaling, PGC-1α and PRC while leading to decreases in PGC-1β mRNA expression. Likewise, a 12-hour treatment with IL-6 (75 ng/ml) resulted in small, but significant increases in the expression of the mitochondrial enzymes COXIV (Cytochrome C Oxidase Subunit IV) and CPT-1 (Carnitine palmitoyltransferase 1) (Figure 1B). In contrast to epididymal adipose tissue, IL-6 at the dosage used, did not induce the expression of SOCS3, PGC-1α, β or PRC in subcutaneous adipose tissue (Figure 1C). The protein content of IL-6 receptor alpha and GP130 (glycoprotein 130) were reduced in subcutaneous compared to epididymal adipose tissue (Figure 1D).

### Mitochondrial proteins are not reduced in adipose tissue from IL-6^−/−^ mice

**Figure 2:**
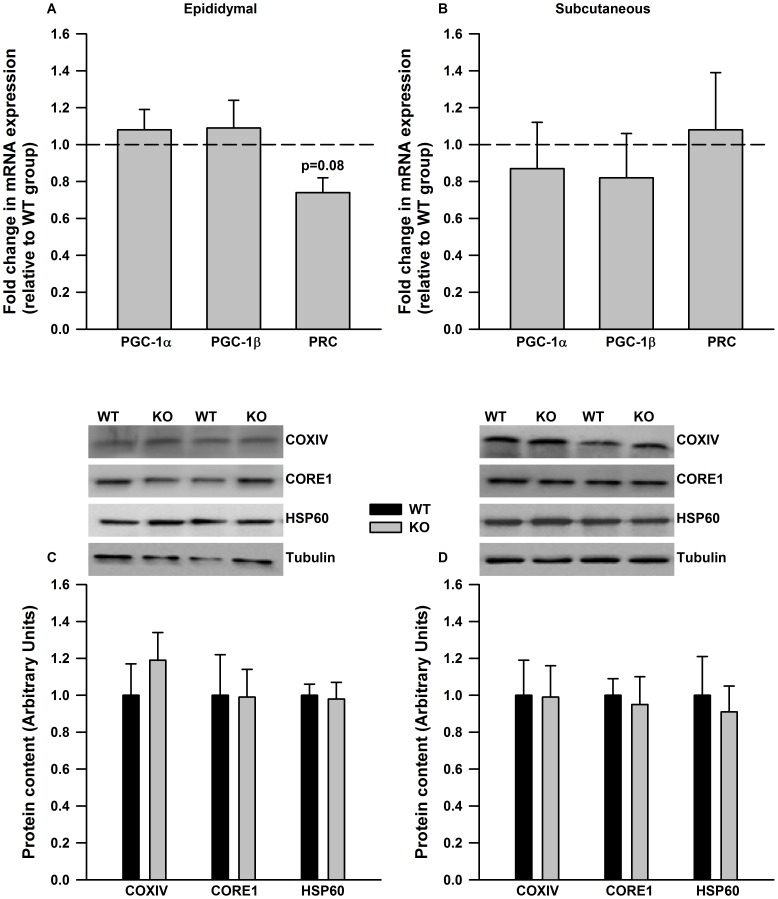
PGC-1 expression and mitochondrial markers proteins are not different in adipose tissue from WT and IL-6^−/−^ mice. The mRNA expression of PGC-1 co-activators (A and B) and mitochondrial marker proteins (C and D) are not different in epididymal (A and C) and subcutaneous adipose tissue (B and D) from WT and IL-6^−/−^ deficient mice. In A and B mRNA data is expressed relative to values in WT controls. Representative Western blots are given above the quantified data in C and D. Data are means + SE for 10 samples per group.

Having shown a direct effect of IL-6 on the induction of PGC-1α, PRC and mitochondrial enzymes we next wanted to determine if the ablation of IL-6 led to reductions in mitochondrial proteins in adipose tissue in vivo. The mRNA expression of PGC-1α and PGC-1β were not different in epididymal adipose tissue from WT and IL-6^−/−^ mice whereas there was a trend (p = 0.08) for a reduction in PRC (Figure 2A). There were no differences in the expression of PGC-1 co-activators in subcutaneous adipose tissue (Figure 2B) from WT and IL-6^−/−^ mice. Similarly, the protein content of the mitochondrial marker proteins CORE1 (Ubiquinol-Cytochrome C Reductase), COXIV and HSP60 (Heat Shock Protein 60) were similar between genotypes in both epididymal (Figure 2C) and subcutaneous (Figure 2D) adipose tissue.

### The greater insulin resistance in IL-6^−/−^ mice fed a HFD is not explained by differences in adipose tissue mitochondrial content

**Figure 3:**
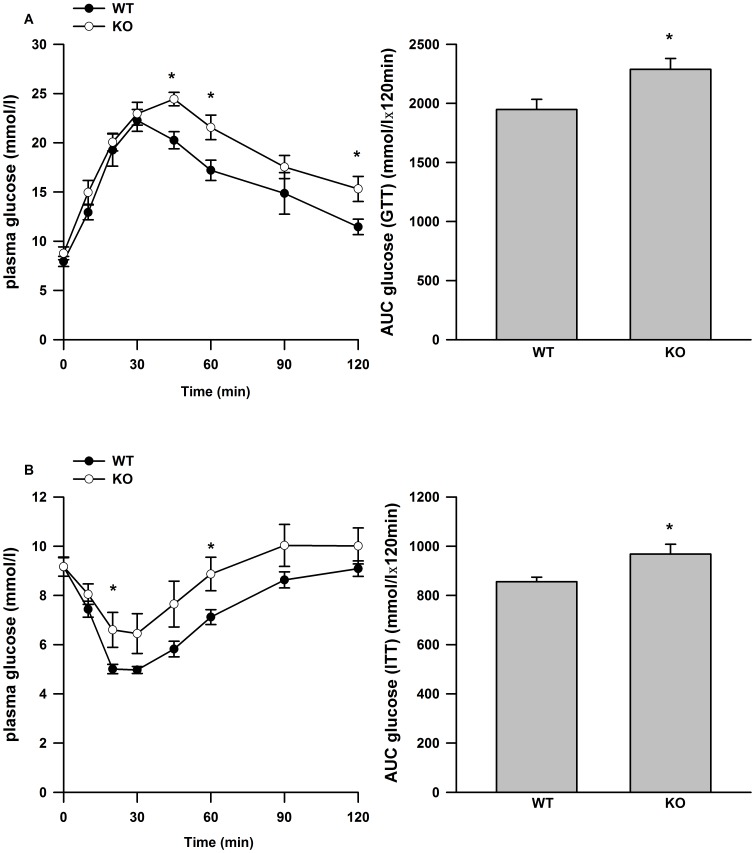
Glucose and insulin tolerance in WT and IL-6^−/−^ mice fed a HFD. Glucose curves and the quantified area under the curves for glucose (A, B) and insulin (C, D) tolerance tests. Data are presented as means +SE for 8–10 mice per group. * P<0.05 versus WT group at the same time point in A and C. * P<0.05 versus WT group in B and D.

**Figure 4:**
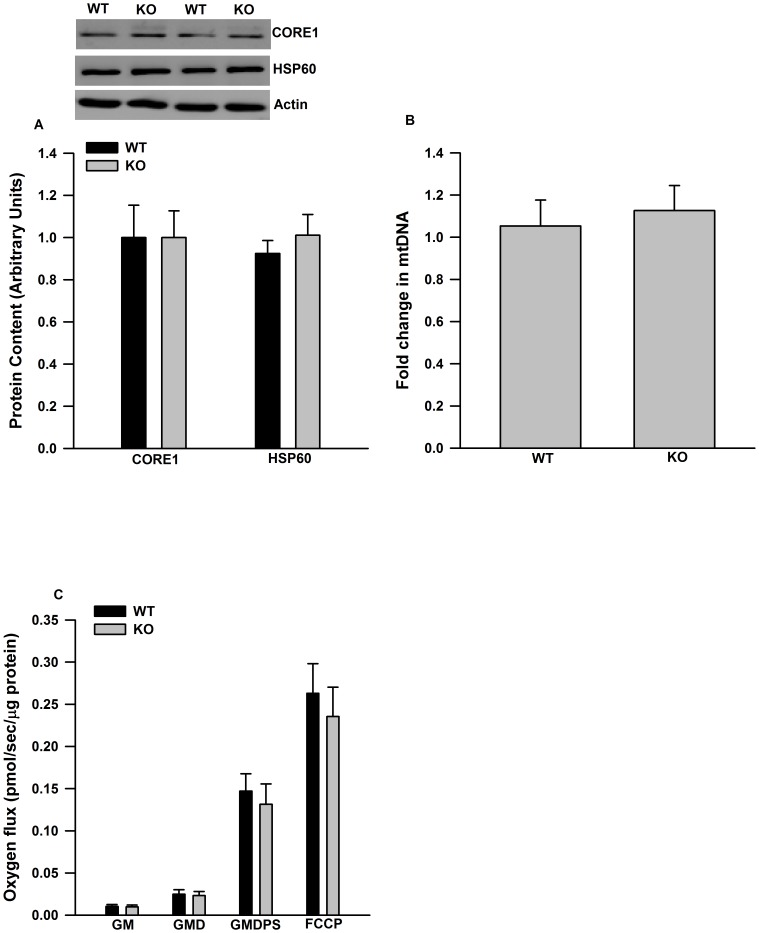
Mitochondrial content and respiration are not altered in epididymal adipose tissue from IL-6^−/−^ mice fed a HFD. A) Mitochondrial marker proteins, B) mtDNA content and C) mitochondrial respiration are similar between genotypes. Data are presented as means + SE for 8–10 animals per group. Western blot images are given above the quantified data in A. Relative mtDNA content is normalized to genomic MCT1 (monocarboxylate transporter 1) DNA content and expressed as fold differences compared to the WT group in B. Mitochondrial respiration was measured by high-resolution O2 consumption. GM (glutamate, malate), GMD (glutamate, malate, ADP), GMDPS (glutamate, malate, ADP, pyruvate, succinate).

IL-6 deficient mice fed a high fat diet (HFD) for 10 weeks were more glucose (Figure 3A) and insulin (Figure 3B) intolerant than WT mice fed a HFD. There were no differences in body weight or epididymal fat pad mass between genotypes (Table 1). In wild type mice, the consumption of HFD led to an increase in the mRNA expression of IL-6 in epididymal adipose tissue compared to chow fed controls (data not shown). The content of mitochondrial marker proteins (Figure 4A) and mitochondrial DNA (Figure 4B) were similar in epididymal adipose tissue from WT and IL-6 deficient mice fed a HFD. Similarly, mitochondrial respiration through complex I (glutamate and malate), complex I and II (glutamate, malate, pyruvate and succinate) and uncoupled respiration were similar between genotypes (Figure 4C).

**Table 1:** Descriptive characteristics of WT and IL-6^−/−^ mice fed a high fat diet (60% Kcals from fat) for 10 weeks (N = 9–10/group).

	WT	KO	p-value
Initial body weight (g)	27.6±0.5	27.4±0.4	0.80
Final body weight (g)	41.8±1.4	44.2±1.0	0.09
Epididymal fat pad weight (g)	1.85±0.41	2.14±0.15	0.44
Average food intake (g per week)	18.90±0.4	18.7±0.7	0.78

## Discussion

Accumulating evidence has highlighted the importance of adipose tissue mitochondria in the control of not only adipose tissue insulin action, but systemic glucose homeostasis as well [9], [33], [34]. We [21], and others [32], have shown that IL-6 regulates the activity of AMPK in adipose tissue. As AMPK is a reputed mediator of PGC-1α [22], we thought it likely that IL-6 would regulate mitochondrial enzymes in adipose tissue. To address this question we assessed the ability of IL-6 to directly induce PGC-1 co-activators and mitochondrial enzymes in cultured adipose tissue and examined changes in PGC-1 and markers of mitochondrial content in adipose tissue from whole body IL-6^−/−^ mice.

We found that IL-6 increased the expression of PGC-1α, PRC and mitochondrial enzyme gene expression in cultured epididymal adipose tissue, while leading to reductions in the expression of PGC-1β. As PGC-1α and β are thought to control an overlapping set of genes [2], [3], the opposite regulation of these transcriptional co-activators is somewhat surprising and would suggest that distinct mechanisms are involved in the regulation of PGC-1α and β in adipose tissue. For example ERRγ (Estrogen Related Receptor Gamma) has been shown to regulate PGC-1β in adipocytes [35], whereas ERRα has been implicated in the control of PGC-1α [36]. Although speculative, and requiring further investigation, this could be a potential mechanism to explain the divergent effects of IL-6 on PGC-1α and β in adipose tissue.

In contrast to epididymal adipose tissue, IL-6, at least at the dosage and duration used in our studies, did not increase the expression of SOCS3, a marker of the activation of IL-6 signaling [37] in subcutaneous adipose tissue. The absence of a detectable effect of IL-6 in cultured subcutaneous adipose tissue was associated with a lower expression of proteins that are involved in the IL-6 receptor signaling complex including GP130 and the alpha subunit of the IL-6 receptor [38]. These results highlight clear differences in IL-6 responsiveness between depots, a finding complementing previous work reporting greater IL-6 secretion from abdominal versus subcutaneous adipose tissue [18].

Given the direct effects of IL-6 on the induction of PGC-1α, PRC and mitochondrial enzyme gene expression we hypothesized that markers of mitochondrial content and/or function would be reduced in adipose tissue from IL-6 deficient mice. Surprisingly we found that PGC-1α and mitochondrial marker proteins were similar in epididymal adipose tissue between genotypes. These results are in contrast to previous reports which demonstrated reductions in PGC-1α mRNA expression and mitochondrial proteins in skeletal muscle [39] and liver [25] from IL-6^−/−^ mice. In contrast to PGC-1α, we found that there was a trend (p = 0.08) towards a reduction in the expression of PRC in adipose tissue from IL-6^−/−^ mice. As IL-6 directly increased the expression of PRC in cultured adipose tissue these findings would perhaps suggest a role for IL-6 in the control of PRC expression in adipose tissue in vivo. Further work is needed to more definitively examine the role of IL-6 in regulating PRC, and in turn identifying the metabolic networks that PRC controls in adipose tissue.

As in any knockout model, the observed phenotype should be viewed cautiously as compensatory increases in redundant signaling pathways could mask the true effect of the deleted gene. With this point in mind, it is plausible that other signaling pathways that are involved in the regulation of mitochondrial biogenesis could be up-regulated in adipose tissue from IL-6 deficient mice. In a recent study from our laboratory [21] we have shown that the activation of p38 MAPK (p38 mitogen activated protein kinase) and CREB (cAMP response element binding protein), which are reputed mediators of mitochondrial biogenesis and PGC-1 in adipocytes [40], [41], are similar in adipose tissue from WT and IL-6^−/−^ mice. Moreover, the responsiveness to epinephrine, a hormone we have shown induces PGC-1α mRNA [42], is also similar between genotypes. Lastly, the expression of PGC-1β, which has been reported to induce mitochondrial genes in 3T3 adipocytes [43], is not elevated in adipose tissue from IL-6^−/−^ mice. Taken together this data demonstrates that there is no obvious up-regulation of signaling pathways involved in mitochondrial biogenesis in adipose tissue from IL-6^−/−^ mice that could be compensating for the lack of IL-6. While we can not discount the possibility that additional, yet to be identified pathways, involved in adipose tissue mitochondrial biogenesis could be up-regulated, we interpret our findings as suggesting that IL-6 is sufficient to induce PGC-1α, and mitochondrial enzyme gene expression ex vivo, but is not necessary for the maintenance of adipose tissue mitochondrial content in vivo.

Although not a universal finding [44], IL-6^−/−^ mice have been shown to develop age-associated insulin resistance [25], [26] and when fed a HFD become markedly more insulin resistant than WT controls [25]. IL-6 expression is elevated in adipose tissue from mice fed a HFD [31] and given the direct effects of IL-6 on the induction of genes involved in mitochondrial biogenesis, we thought it plausible that studying mice under the conditions of a metabolic challenge of a HFD, could uncover a role for IL-6 in the regulation of adipose tissue mitiochondrial content. We hypothesized that reductions in adipose tissue mitochondrial content and/or respiration may explain, at least in part, the greater insulin resistance present in IL-6 deficient mice fed a HFD. In keeping with recent work from Febbraio's group [25], we found that IL-6 deficient mice were more glucose and insulin intolerant than WT controls when fed a HFD, demonstrating that IL-6 may play a protective role against the development of insulin resistance in conditions of chronic nutrient excess. However the reported differences in glucose homeostasis were not explained by reductions in adipose tissue mitochondrial content or respiration in IL-6^−/−^ mice. The disconnect between impaired glucose homeostasis and reductions in mitochondrial content is consistent with previous work from our laboratory demonstrating that reductions in adipose tissue mitochondrial content in rats fed a HFD is not a causative event in the development of impaired glucose homeostasis [10]. Similar results have also been reported in Otsuka Long-Evans Tokushima Fatty rats [45]. The fact that reductions in adipose tissue mitochondrial content and function are dissociated from differences in glucose homeostasis in three distinct models questions the importance of adipose tissue mitochondrial “dysfunction” in the etiology of systemic insulin resistance.

In summary, we have shown depot-specific differences in the response of mouse adipose tissue to IL-6 ex vivo. We have further demonstrated that IL-6, although sufficient to induce PGC-1α, PRC and mitochondrial enzyme gene expression is not required for the maintenance of adipose tissue mitochondrial content in lean animals, or in mice fed a HFD. Lastly, the greater insulin resistance in IL-6 deficient mice fed a HFD is not explained by differences in adipose tissue mitochondrial content. Taken in context with previous work from our laboratory [10], and others [45], the results of the current study demonstrate that it is unlikely that adipose tissue mitochondrial “dysfunction” is involved in the pathogenesis of systemic insulin resistance.

## Methods and Materials

### Materials

Reagents, molecular weight marker and nitrocellulose membranes for SDS-PAGE were purchased from Bio-Rad (Mississauga, ON). ECL Plus was a product of Amersham Pharmacia Biotech (Arlington Heights, IL). Antibodies against CORE1 (CAT# MS303) and COXIV (CAT# MS407) were obtained from Mitosciences (Eugene, OR) while HSP60 antibodies (CAT# SPA-807) were from Enzo Life Sciences (Ann Arbor, MI). GP130 (CAT# 3732) antibodies were purchased from Cell Signaling (Danvers, MA). Antibodies against tubulin (CAT#ab7291) were from Abcam (Cambridge, MA). An antibody against IL-6 receptor alpha (CAT# SC-660) was obtained from Santa Cruz Biotechnology (Santa Cruz, CA). Horseradish peroxidase-conjugated donkey anti-rabbit and goat anti-mouse IgG secondary antibodies were purchased from Jackson Immuno-Research Laboratories (West Grove, PA). SuperScript II Reverse Transcriptase, oligo(dT) and dNTP were products from Invitrogen (Burlington, ON). Taqman Gene Expression Assays for mouse β actin (4352933E), PGC-1α (CAT# Mm01208835_m1), PGC-1β (CAT# Mm00504720_m1), PRC (CAT# Mm00521078_m1), COXIV (CAT# Mm01250094_m1), CPT-1 (CAT # Mm01308166_m1) and IL-6 (CAT# Mm00446190_m1) were from Applied Biosytems (Foster City, CA). The high fat diet was purchased from Harlan Laboratories Inc. (CAT# TD06414, Madison, WI). All other chemicals were purchased from Sigma (Oakville, ON).

### Treatment of animals

All protocols followed Canadian Council on Animal Care (CCAC) guidelines and were approved by the University of Guelph Animal Care Committee. Twelve-week-old male IL-6^−/−^ mice (Jackson Laboratories B6.12952-IL6^tmlkopf^/J) and age-matched C57BL/6J wild-type (WT) mice were housed 2 per cage, with a 12/12-hour light/dark cycle and were fed standard rodent chow ad libitum. For the high fat diet experiments 13 week old WT and IL-6^−/−^ mice were fed a diet containing 18.4% protein (casein), 60.3% fat, and 21.3% carbohydrate ad libitum for 10 weeks.

### Glucose and Insulin Tolerance Tests

Intraperitoneal (I.P.) glucose (GTT) and insulin tolerance tests (ITT) were performed as an assessment for whole body glucose homeostasis. For the GTT, mice were fasted for 6 h prior to an I.P. injection of glucose (2 g/kg body weight). For the ITT, mice had free access to food prior to an I.P. injection of insulin (0.75 U/kg body weight). Blood glucose levels were determined by tail vein sampling at the indicated intervals using a glucometer. Changes in glucose over time were plotted, and the area under the curve (AUC) was calculated for each.

### Adipose Tissue Organ Culture

Adipose tissue organ culture is a well characterized technique that has been used to determine changes in adipose tissue metabolism and gene expression [46]. Epididymal and inguinal subcutaneous adipose tissue from chow fed C57BL/6J mice were cultured as we have described in detail previously [42], [47]. Briefly, mice were anesthetized with pentobarbital sodium (5 mg/100 g body wt) and epididymal and inguinal subcutaneous adipose tissue were removed, weighed and immediately placed in 50 ml conical tubes containing sterile PBS with 1% antibiotic/antimycotic. Under sterile conditions, ∼100 mg of tissue was placed into culture dishes containing 3 ml of M199 supplemented with 1% antibiotic/antimycotic, 50 µU insulin and 2.5 nM dexamethasone added to the media. The tissue was then minced into ∼5–10 mg pieces and kept in a cell incubator at 37°C to equilibrate for 24 h. To assess the effects of IL-6 on the mRNA expression of PGC-1α, PGC-1β, and PRC adipose tissue was treated with mouse recombinant IL-6 (75 ng/ml) for 6 hrs. To determine the effects of IL-6 on the induction of COXIV, a respiratory chain enzyme, and CPT-1, an enzyme involved in mediating the entry of fatty acids into the mitochondria, tissue cultures were treated with 75 ng/ml IL-6 for 12 hours. At the end of the experiments adipose tissue cultures were rinsed in ice-cold sterile PBS, strained and adipose tissue fragments snap frozen and stored at −80° C for further analysis.

### Mitochondrial respiration in adipose tissue

High-resolution O_2_ consumption measurements were conducted in 2 mL of Miro5 using the OROBOROS Oxygraph-2k (OROBOROS INSTRUMENTS, Corp., Innsbruck, AT) with stirring at 750 rpm at 37°C. Epididymal adipose tissue was removed from WT and IL-6^−/−^ mice and ∼40–50 mg were minced with scissors (∼30 seconds) and placed in an Oxygraph-2k reaction chamber. In agreement with a previous report [48], preliminary experiments demonstrated no additional effect of standard membrane permeabilization detergents (digitonin, saponin) on respirometric assessments. This suggests the surgical and mincing procedures are sufficient for maximal respiration in adipose tissue.

Adipose samples were allowed to incubate for ∼10 min before initiating the respirometric protocols. 10 mM glutamate (G) and 5 mM malate (M) were then added as complex I substrates (State IV respiration) followed by 5 mM ADP (D; State III respiration). Additional electron entry into complex I and II were achieved with 10 mM pyruvate (P) and 20 mM succinate (S) respectively. ETS capacity was determined via titration of FCCP to induce maximal uncoupled respiration (∼typically occurred with 0.5 µM FCCP). All experiments were completed before oxygraph chamber [O_2_] reached 100 µM, which is above the threshold of O_2_ limitation we have observed in adipose tissue (∼60–80 µM). Polarographic oxygen measurements were acquired in 2-second intervals, with the rate of respiration derived from 40 data points, and expressed as pmol • s^−1^ • µg^−1^ protein at 37°C. Cytochrome *c* was added to test for mitochondrial membrane integrity and respiration was never increased by >10%.

### Western Blot Analysis

Protein was extracted from adipose tissue and the content of COXIV, CORE1, HSP60, GP130 and IL-6Rα determined by Western blotting as described in detail by our laboratory previously [42], [47]. Signals were detected using enhanced chemiluminesence and were subsequently quantified by densitometry by Gene Tool according to the manufacturer's instructions (SynGene, ChemiGenius2, PerkinElmer).

### Real Time PCR

RNA was isolated from adipose tissue using an RNeasy kit according to the manufacturer's instructions. 1 µg of RNA was used for the synthesis of complementary DNA (cDNA) using SuperScript II Reverse Transcriptase, oligo(dT) and dNTP. Real time PCR was performed using a 7500 Fast Real-Time PCR system (Applied Biosytems), as described previously [49]. Each assay (20 µl total volume) contained 1 µl gene expression assay, 1 µl of cDNA template, 10 µl of Taqman Fast Universal PCR Master Mix and 8 µl of RNase free water. For β-actin or 18S, each 50 µl reaction contained 25 µl of PCR Master mix, 2.5 µl each of gene expression assay, 1 µl of cDNA template, and 21.5 µl of RNase free water. Results were normalized to the mRNA expression of β-actin for the adipose tissue organ culture experiments or 18S for samples procured from WT and IL-6^−/−^ mice. Ex-vivo IL-6 treatment did not alter the mRNA expression of beta actin in cultured subcutaneous (vehicle 19.28±0.35, 6 hr IL-6 19.24±0.42 raw CTs) or epididymal (vehicle 17.92±0.16, 6 hr IL-6 17.90±0.33 raw CTs) adipose tissue. Likewise, the expression of 18s in epididymal (WT 15.75±0.12, IL-6^−/−^ 15.94±0.08 raw CTs) and subcutaneous (WT 15.91±0.16, IL-6^−/−^ 15.83±0.15 raw CTs) adipose tissue from chow fed WT and IL-6^−/−^ mice were similar, as was the expression of 18s in epididymal adipose tissue from WT (15.88±0.15) and IL-6^−/−^ (15.86±0.35) mice fed a high fat diet. Relative differences in gene expression between groups were determined using the 2^−ΔΔCT^ method [50]. The amplification efficiencies of the gene of interest and the housekeeping gene were equivalent.

### Mitochondrial DNA

Total DNA was isolated using DNeasy blood and tissue kit (Qiagen) and relative mitochondrial DNA copy number was determined as described in detail previously by Holloway et al [51].

### Statistical Analysis

Comparisons between two groups were made using a Students T-Test. Statistical significance was established at a P level<0.05.
